# Order scheduling optimization in manufacturing enterprises based on MDP and dynamic programming

**DOI:** 10.1038/s41598-023-36976-7

**Published:** 2023-06-16

**Authors:** Qiqige Wulan

**Affiliations:** Management Science and Engineering Department, Dongbei University of Finance and Economics, Dalian, 116025 China

**Keywords:** Applied mathematics, Computational science, Computer science

## Abstract

In the era of Industry 4.0, order scheduling is a crucial link in the production of manufacturing enterprises. In view of order scheduling in manufacturing enterprises, a finite horizon Markov decision process model is proposed in this work based on two sets of equipment and three types of orders with different production lead times to maximize the revenue in manufacturing production systems. Then, the dynamic programming model is incorporated into the optimal order scheduling strategy. Python is employed to simulate the order scheduling in manufacturing enterprises. Based on survey data, the superiority of the proposed model compared to traditional first come, first served order scheduling is verified by experimental cases. Finally, sensitivity analysis is conducted on the longest service hours of the devices and the order completion rate to explore the applicability of the proposed order scheduling strategy.

## Introduction

In the era of Industry 4.0, manufacturing enterprises with limited production capacity must make reasonable order scheduling and scientific production arrangements to meet growing customer demands. In this way, they can fully utilize the existing production capacity and ensure mass production and timely delivery, effectively shortening the order lead time, reducing order delay, and maximizing customer demand satisfaction^1–3^. Unscientific order scheduling substantially slows the development of enterprises. On the one hand, an excessive demand for orders cannot be met with the conventional production capacity of a production line, so enterprises must address this problem through other means. Meanwhile, the overtime production of enterprises is prone to excessive equipment load, shortening the working life of machines and affecting the normal operation of the enterprise. Subcontracting orders increases costs and reduces profits and is not conducive to the long-term development of the enterprise. If enterprises improve their production capacity by increasing or upgrading equipment, the demand for orders can be satisfied, but this approach increases the fixed-variable cost and the business risk of the enterprises. In the postpandemic age of 2022, it is even more important for enterprises to respond flexibly and efficiently to rapidly changing needs and new challenges. An increasing number of enterprises believe that blindly increasing production capacity will result in a fatal blow to development. On the other hand, insufficient order scheduling will lower the current risks of the enterprise, but it may cause a waste of production resources, which will result in reduced profits, lost customers, and impairment of long-term development. Therefore, formulating a set of order scheduling strategies that comprehensively considers the economic benefits and long-term development of the enterprise and solves the production capacity fluctuation caused by the "imbalance of busy and idle time" of the production line is important for current manufacturing enterprises^4^.

Generally, the orders of manufacturing enterprises are based on various customer needs and can usually be classified as follows: standard orders, which have a standard bill of material with a fixed lead time and stock; nonstandard orders, which refer to customized products without BOMs; and emergency orders, which are short delivery requests. Different types of orders reflect various demand characteristics of customers. Standard orders and nonstandard orders give the manufacturing enterprises specific lead times, while emergency orders come randomly on the day of production scheduling. Emergency orders are urgent, regardless of cost. Usually, the customer will bear the component transportation costs and emergency service costs, or the enterprise must accept a large delay penalty due to delayed delivery time. Therefore, an emergency order should be arranged for production immediately when it is accepted; otherwise, it will cause a higher rejection cost. The customer required delivery (CRD) for standard and nonstandard orders is relatively relaxed, allowing for reasonable waiting during the specified lead time. In addition, different types of orders require different production times in practice^5–8^.

There are two major tasks for the effective management of production scheduling in manufacturing enterprises. One is to create JIT (just-in-time) production scheduling to respond to customer demand, that is, the delivery time and ineffective waiting can be reduced by placing orders in advance. The other is to determine the decision rules for MRP (material requirements planning) of the production scheduling system, based on which the ERP system can decide to accept or reject orders. At present, most manufacturing enterprises adopt ERP systems to assign customer orders to the latest idle time of the production equipment based on the FCFS (first come, first served) principle. Such production scheduling fails to consider the demand characteristics of different types of orders. Meanwhile, a factory is likely to reject orders that arrive later but have higher returns due to a lack of production equipment and insufficient production capacity^9–11^. Targeting the above deficiencies, an MDP (Markov decision process) model in the finite time domain^12^ is established and combined with dynamic programming theory to analyze the optimal scheduling of limited production equipment resources among different types of orders to maximize the production benefits of manufacturing enterprises.

## Literature review

Order scheduling of manufacturing enterprises is an extensive research topic worldwide. Practical production and manufacturing are affected by various factors, such as the arrival of emergency orders, order cancellation, and raw material interruption. Thus, static production and production scheduling face great difficulties in practice; therefore, dynamic production scheduling is increasingly implemented. Nie et al.^13^ studied dynamic single-machine order production scheduling with workpiece release time and proposed a production scheduling rule constructor based on gene expression programming to construct effective production scheduling rules. Targeting high efficiency and stability, Fat et al.^14^ studied the dynamic production scheduling of flexible operation workshops, proposed a metaheuristic algorithm based on the genetic algorithm, and achieved effective computational experimental results. Vinod et al.^15^ simulated the interaction between the delivery time distribution method and production scheduling rules in the typical production system of a dynamic operation workshop in various application scenarios. Pickardt et al.^16^ proposed a two-stage hyperheuristic algorithm by combining the genetic planning algorithm with the evolutionary algorithm to generate a set of production scheduling rules to solve complex dynamic production scheduling in an operation workshop in the semiconductor manufacturing industry. Hamzadayi et al.^17^ proposed fully reactive order scheduling based on the simulated annealing algorithm and allocation rules and optimized the dynamic production scheduling of multiple equivalent parallel machines controlled by a single server using an event-driven production scheduling strategy. Rajabinasab et al.^18^ investigated the dynamic production scheduling of a class of flexible operation workshops and considered dynamic events (such as the random arrival of workpieces, uncertain processing time, accidental machine failure, and path and process flexibility). Then, they developed a multiagent production scheduling system and obtained an efficient and robust production scheduling strategy. Zhou et al.^19^ proposed a task scheduling method in a dynamic cloud manufacturing environment with random arrival tasks. Based on MDP theory, Wang et al.^20^ optimized a resource scheduling strategy in the case of dynamic change. Through mathematical modeling and software solutions, they identified the best scheduling strategy under specific conditions, ensuring timeliness. On the basis of traditional production scheduling, Qian et al.^21^ proposed a more complete model for the order acceptance of MTO (make-to-order) enterprises. Meanwhile, they considered the inventory cost of orders and various customer priority factors based on the delayed delivery cost, rejection cost, production cost, and other traditional model elements. Finally, they modeled the optimal production scheduling decision as an MDP. Related Scheduling Strategy and Algorithm is as below Tables 1 and 2.

**Table 1:** Classic scheduling strategy.

Algorithm	Core ideas	Advantage	Disadvantage
Minimum completion time	Assign tasks to the resource with the smallest completion time in any order	Short completion time for single task	Long overall completion time
First come, first served (FCFS)	Allocate resources in the order in which tasks arrive	Simple implementation	The average waiting time is very long
Round Robin	Rotate tasks from users to resources each time	Simple and efficient	Easy to cause load imbalance
Greedy	Making what currently appears to be the best choice	Simple and efficient	Unable to obtain optimal solution with aftereffect
Min–Min	Assign tasks with the minimum completion time to resources with the earliest completion time	Simple and fast	Low load balancing performance
Max–Min	Assign tasks with the highest completion time to resources with the earliest completion time	Simple and fast	Lack of extension

**Table 2:** Improved heuristic algorithm strategy.

Algorithm	Task type	Algorithm improvement points	Optimization objectives	Effect
Improved genetic algorithm	Task scheduling	Coding and decoding, fitness function, Cross mutation	Time, cost, CPU, memory, and bandwidth	Time and cost constraints have been achieved good effect
Improved Differential Evolution Algorithm	Task scheduling	Introduced adaptive scaling factor and crossover operator	Completion time and execution energy consumption	Effectively avoiding falling into local optimal solution and the Defects of Solving and ‘Premature’
Improved Particle Swarm Optimization Algorithm	Task scheduling	Dynamic based on sine improvement strategy Inertia weight	Completion time	Improved the efficiency of task scheduling
Improved Ant Colony Algorithm	Task scheduling	Utilize the state of virtual machines to adapt self-adjust of information	Service stability, resource utilization, Task completion time	Can also quickly find the most Optimal solution during handling large-scale task assignments
Genetic Ant Colony Algorithm	Task scheduling	First, use genetic algorithm to find the optimal solution, then use ant colony algorithm to find Global optimal solution	Completion time, bandwidth, reliability, cost	Fast convergence speed
Cuckoo particle swarm algorithm	Task scheduling	Two types of calculations are used in the solving process to obtain a new solution	Completion time, cost, deadline, Violation rate	Fast convergence speed and easy implementation
Improved ant colony algorithm	Resource scheduling	Introducing sequential exchange and migration operations	Resource utilization of servers	In obtaining better solutions and faster convergence speed performance
Improved grouping genetic algorithm	Resource scheduling	Using the optimal random insertion algorithm and worst case random deletion algorithm for optimization	Resource utilization rate and energy consumption	Can quickly adopt the least amount of physics Machine to place virtual machines
Improving the memetic algorithm	Resource scheduling	Improved the performance of the memetic algorithm partial search strategy	Energy consumption and runtime service level coordination, negotiate violation rate and resource utilization rate	Reduced Time cost for placing issues during algorithm application to virtual machines

At present, there are many different research directions and solution methods for order scheduling and production scheduling in manufacturing enterprises, laying a foundation for further research. The current literature on production line optimization order scheduling fails to fully consider the characteristics of different types of customer orders because the model establishment and solution become complex with the increase in consideration factors. There are still a few studies on multiple production line equipment, and the different production times for various types of orders are rarely included. However, differences in the order production time affect the optimal scheduling of the production capacity. This paper considers two production lines with three types of orders and their different production times. Based on reasonable hypotheses, an MDP in the finite time domain is established, and the optimal production scheduling strategy for orders is obtained using Python. Then, the applicability of the proposed production scheduling strategy is checked through sensitivity analysis.

## Model description

This paper studies the optimal production scheduling strategy for two production lines and three types of production orders requiring different production times and revenues. Production scheduling for manufacturing enterprises aims to maximize the benefits in terms of production equipment. When an order arrives, the PRP system immediately decides whether to accept it according to the decision rules and offers a reasonable production schedule in accordance with the set production scheduling rules.

### Model assumption

#### Assumption (H1)

Production line equipment A and equipment B, which are no different in terms of service condition and production capacity, are allowed to produce an order simultaneously.

#### Assumption (H2)

The production capacity of the production equipment is measured by the time slot, which can be set as the base of time according to the actual situation, and the longest service hours per day for equipment A and equipment B are the same.

#### Assumption (H3)

The production times required by different types of orders are different and can be represented by the number of time slots. The inspection time of nonstandard orders is longer than the production time for standard orders (usually due to more processes).

#### Assumption (H4)

The entire production cycle can be divided into a limited number of equal moments. At any moment, the production scheduling requests of standard orders and nonstandard orders are entered into the system with different probabilities, which are independent of each other. Emergency orders are arranged randomly on a given day of the production line.

#### Assumption (H5)

At any moment, only one order production request is entered into equipment at any production time, that is, there are at most two production orders at any production time.

#### Assumption (H6)

The order arrangements of equipment A and equipment B are independent of each other. At any production time, there are only three possibilities for each piece of equipment, namely, a production request for a standard order, a nonstandard order, or a random emergency order.

#### Assumption (H7)

At any moment, the production scheduling rules of the system are set as follows. If the system accepts a production request for only one order (a standard order or a nonstandard order), the equipment with a larger surplus capacity is preferred. If the surplus capacities of both equipment are equal, equipment A is selected. If the system accepts a production request for a standard order and a nonstandard order, it will give priority to the equipment with more surplus capacity for nonstandard order production. If the system accepts production requests for two orders of the same type (two standard orders or two nonstandard orders), they are assigned to equipment A and equipment B separately.

**Figure 1:**
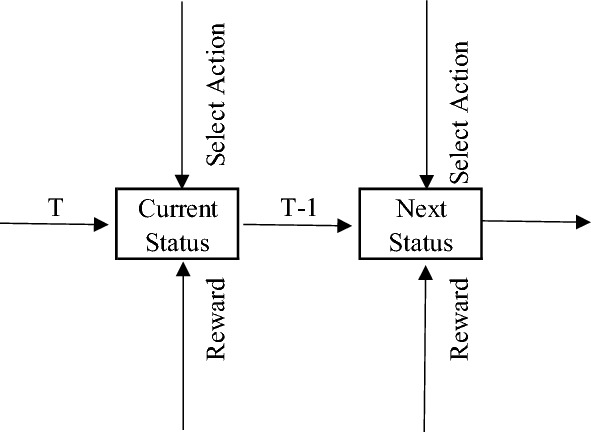
Decision-making process.

Based on the above assumptions, scheduling of the production line can be described using an MDP in the finite time domain, and maximizing the enterprise revenue can be transformed into obtaining the optimal solution of dynamic programming. The MDP refers to the decision maker periodically or continuously observing a random dynamic system of a Markov process and making decisions sequentially. In the MDP, the system model depends on only the current state and selected strategy rather than the historical state and strategy. The MDP model can make the optimal decision satisfying the conditions at any production time based on the system state and the order arrangement, as shown in Fig. 1. The MDP can be described by a quintuple:

$${\text{M}} = \left\langle {{\text{T}},{\text{S}},{\text{A}},{\text{P}},{\text{R}}} \right\rangle$$

where T, S, and A are the set of decisions, system states, and available actions, respectively; P is the state transition probability, P(s′|s, a) P(s′|s, a) indicates the probability that taking action “a” from state “s” reaching state “s”; R is the reward function, and R(s,a) represents the immediate reward for executing action a at state S.

### Model parameter settings

In this paper, the maximum number of available time slots for equipment A and equipment B each day is assumed to be one; then, the total number of available time slots for the production line is one per day. The whole production cycle can be divided into many equal production moments, and all moments in the production cycle are expressed as t = T, t − 1… 2, 1. The production request of an order arrives during one of these moments with a certain probability. t = T and t = 0 are the beginning and end of the production cycle, respectively. The other parameter settings are listed in Table 3.

**Table 3:** Model parameter settings.

Parameter	Definition
e	The number of time slots during which the production line produces a standard order
f	The number of time slots during which the production line produces a nonstandard order
g	The number of time slots during which the production line produces an emergency order
λ1	The probability that a standard order reaches the production system
λ2	The probability that a nonstandard order reaches the production system
Q	The number of emergency orders that randomly arrived on a production day
π_1_	The income obtained from a standard order
π_2_	The income obtained from a nonstandard order
π_3_	The income obtained from an emergency order
c_1_	The penalty costs caused by rejecting a standard order
c_2_	The penalty costs caused by rejecting a nonstandard order
c_3_	The penalty costs caused by rejecting an emergency order
c_4_	The idle cost per unit time slot of the production line on the current day
T_A_	At each production time, the number of remaining time slots of equipment A, 0 ≤ TA ≤ T
T_B_	At each production time, the number of remaining time slots of equipment B, 0 ≤ TB ≤ T

### Model establishment

According to the five elements of the MDP (decision stage, state set, decision set, transfer probability, and reward), the MDP model in the production cycle is established, and the objective function is solved.

#### Decision stage T

It refers to any production time T in the production cycle, that is, T = T, t − 1… 2, 1.

#### State set S

TA and TB represent the numbers of available time slots for equipment A and equipment B, respectively. S = (TA, TB) is the set of states of the system, satisfying 0 ≤ TA ≤ T and 0 ≤ TB ≤ T.

#### Decision set A

At each production moment, t = T, T−1…, 2, 1, the system makes a decision based on the current number of remaining time slots and the arrangement of the order, that is, to accept or reject a production request. The action set of the system is represented by At (TA,TB), where 0 and 1 represent the rejection and acceptance of the production request, respectively. Then, the following relationship can be obtained:

$${\text{At}}\left( {\text{TA,TB}} \right) = \left\{ {\begin{array}{*{20}l} 0 \hfill & {\quad {\text{RejectOrder}}} \hfill \\ 1 \hfill & {\quad {\text{AcceptOrder}}} \hfill \\ \end{array} } \right.$$

#### Transfer probability P

There are three possibilities (a standard order, a nonstandard order, and no production task) for the order arrangement of equipment at any production moment. Then, the production system may have six states at any time, which correspond to six different transfer probabilities (Table 4).

**Table 4:** Arrival and transfer probabilities of orders.

Order arrival status	Transfer probability
Two standard order requests arrive	${\text{P}}_{1} = \lambda_{1}^{2}$
Two nonstandard order requests arrive	${\text{P}}_{2} = \lambda_{2}^{2}$
A standard order & a nonstandard order request arrive	P_3_ = 2λ_1_ λ_2_
Only a standard order request arrives	P_4_ = 2λ_1_ (1 − λ_1_ − λ_2_)
Only a nonstandard order request arrives	P_5_ = 2λ_2_ (1 − λ_1_ − λ_2_)
No production request arrives	P_6_ = (1 − λ_1_ − λ_2_)^2^

#### Reward R

The benefits of an enterprise in producing a standard order, a nonstandard order, and an emergency order are π_1_, π_2_, and π_3_, respectively. Accordingly, the costs of rejecting a standard order, a nonstandard order, and an emergency order are expressed as C_1_, C_2_, and C_3_, respectively. Generally, π_1_ + C_1_ < π_2_ + C_2_ + π_3_ + C_3_; that is, the comprehensive income of an emergency order is the highest and that of a standard order is the lowest. In other words, emergency orders should be given the highest priority, and nonstandard orders are generally of higher priority than standard orders.

#### Object function V

Vt (TA,TB) represents the maximum revenue from time t, the current state of the system S = (TA,TB), to the end of the production cycle.

$K_{t}^{i} (T_{A} ,T_{B} )$ represents the maximum income that the enterprise can obtain from the current state of the system S = (TA,TB) at time t (after the arrival of Class i) to the end of the production cycle, where i = 1, 2, 3, 4, 5 and 6, correspond to six different transition probabilities. According to dynamic programming theory, the optimal income of the following system can be established at any time t = T, t − 1… 1.

1 $$V_{t} (T_{A} ,T_{B} ) = \sum\limits_{i = 6}^{6} {P_{i} } R_{t - 1}^{i} (T_{A} ,T_{B} )$$

where i = 1 when the production requests of two standard orders are given;

2 $$R_{t}^{1} = \left\{ \begin{gathered} \max \left\{ \begin{gathered} V_{t} (T_{A} - e,T_{B} - e) + 2\pi_{1} ,V_{t} (T_{A} - e,T_{B} ) + \pi_{1} - c_{1} \hfill \\ V_{t} (T_{A} ,T_{B} ) - 2c_{1} \hfill \\ \end{gathered} \right.,T_{A} \ge e,T_{B} \ge e \hfill \\ \max \left\{ {V_{t} (T_{A} - e,T_{B} ) + \pi_{1} - c_{1} ,V_{t} (T_{A} ,T_{B} ) - \left. {2c_{1} } \right\},T_{A} \ge e,T_{B} < e} \right. \hfill \\ \max \left\{ {V_{t} (T_{A} ,T_{B} - e) + \pi_{1} - c_{1} ,V_{t} (T_{A} ,T_{B} ) - \left. {2c_{1} } \right\},T_{A} < e,T_{B} \ge e} \right. \hfill \\ \max \left\{ {V_{t} (T_{A} ,T_{B} ) - \left. {2c_{1} } \right\},T_{A} < e,T_{B} < e} \right. \hfill \\ \end{gathered} \right.$$

i = 2 when the production requests for two nonstandard orders are given;

3 $$R_{t}^{2} = \left\{ \begin{gathered} \max \left\{ \begin{gathered} V_{t} (T_{A} - f,T_{B} - f) + 2\pi_{2} ,V_{t} (T_{A} - f,T_{B} ) + \pi_{2} - c_{2} \hfill \\ V_{t} (T_{A} ,T_{B} ) - 2c_{1} \hfill \\ \end{gathered} \right.,T_{A} \ge f,T_{B} \ge f \hfill \\ \max \left\{ {V_{t} (T_{A} - f,T_{B} ) + \pi_{2} - c_{2} ,V_{t} (T_{A} ,T_{B} ) - \left. {2c_{2} } \right\},T_{A} \ge f,T_{B} < f} \right. \hfill \\ \max \left\{ {V_{t} (T_{A} ,T_{B} - f) + \pi_{2} - c_{2} ,V_{t} (T_{A} ,T_{B} ) - \left. {2c_{2} } \right\},T_{A} < f,T_{B} \ge f} \right. \hfill \\ \max \left\{ {V_{t} (T_{A} ,T_{B} ) - \left. {2c_{2} } \right\},T_{A} < f,T_{B} < f} \right. \hfill \\ \end{gathered} \right.$$

i = 3 when the production requests of a standard order and a nonstandard order are given simultaneously;

4 $$R_{t}^{3} = \left\{ \begin{gathered} \max \left\{ \begin{gathered} V_{t} (T_{A} - f,T_{B} - e) + \pi_{1} + \pi_{2} ,V_{t} (T_{A} - e,T_{B} - f) + \pi_{1} + \pi_{2} \hfill \\ V_{t} (T_{A} - e,T_{B} ) + \pi_{1} - c_{2} ,V_{t} (T_{A} - f,T_{B} ) + \pi_{2} - c_{1} \hfill \\ V_{t} (T_{A} ,T_{B} ) - c_{1} - c_{2} \hfill \\ \end{gathered} \right.T_{A} \ge f,T_{B} \ge f \hfill \\ \max \left\{ \begin{gathered} V_{t} (T_{A} - f,T_{B} - e) + \pi_{1} + \pi_{2} ,V_{t} (T_{A} - e,T_{B} - f) + \pi_{1} + \pi_{2} \hfill \\ V_{t} (T_{A} - f,T_{B} ) + \pi_{2} - c_{1} ,V_{t} (T_{A} ,T_{B} ) - c_{1} - c_{2} \hfill \\ \end{gathered} \right.T_{A} \ge f,e \le T_{B} < f \hfill \\ \max \left\{ \begin{gathered} V_{t} (T_{A} - f,T_{B} ) + \pi_{2} - c_{1} ,V_{t} (T_{A} - e,T_{B} ) + \pi_{1} - c_{2} \hfill \\ V_{t} (T_{A} ,T_{B} ) - c_{1} - c_{2} \hfill \\ \end{gathered} \right.T_{A} \ge f,T_{B} < e \hfill \\ \max \left\{ \begin{gathered} V_{t} (T_{A} - e,T_{B} - f) + \pi_{1} + \pi_{2} ,V_{t} (T_{A} - e,T_{B} ) + \pi_{1} - c_{2} \hfill \\ V_{t} (T_{A} ,T_{B} - f) + \pi_{2} - c_{1} ,V_{t} (T_{A} ,T_{B} ) - c_{1} - c_{2} \hfill \\ \end{gathered} \right.e \le T_{A} < f,T_{B} \ge f \hfill \\ \max \left\{ {V_{t} (T_{A} - e,T_{B} ) + \pi_{1} - c_{2} ,V_{t} (T_{A} ,T_{B} )\left. { - c_{1} - c_{2} } \right\},e \le T_{A} < f,T_{B} < f} \right. \hfill \\ \max \left\{ \begin{gathered} V_{t} (T_{A} ,T_{B} - f) + \pi_{2} - c_{1} ,V_{t} (T_{A} ,T_{B} - e) + \pi_{1} - c_{2} \hfill \\ V_{t} (T_{A} ,T_{B} ) - c_{1} - c_{2} \hfill \\ \end{gathered} \right.T_{A} < e,T_{B} \ge f \hfill \\ \max \left\{ {V_{t} (T_{A} ,T_{B} - e) + \pi_{1} - c_{2} ,V_{t} (T_{A} ,T_{B} )\left. { - c_{1} - c_{2} } \right\},T_{A} < e,e \le T_{B} < f} \right. \hfill \\ \max \left\{ {V_{t} (T_{A} ,T_{B} )\left. { - c_{1} - c_{2} } \right\},T_{A} < e,T_{B} < e} \right. \hfill \\ \end{gathered} \right.$$

i = 4 when a production request for a standard order is offered;

5 $$R_{t}^{4} = \left\{ \begin{gathered} \max \left\{ {V_{t} (T_{A} - e,T_{B} ) + \pi_{1} ,V_{t} (T_{A} ,T_{B} ) - \left. {c_{1} } \right\},T_{A} \ge e} \right. \hfill \\ \max \left\{ {V_{t} (T_{A} ,T_{B} - e) + \pi_{1} ,V_{t} (T_{A} ,T_{B} ) - \left. {c_{1} } \right\},T_{A} < e,T_{B} \ge e} \right. \hfill \\ \max \left\{ {V_{t} (T_{A} ,T_{B} ) - \left. {c_{1} } \right\},T_{A} < e,T_{B} < e} \right. \hfill \\ \end{gathered} \right.$$

i = 5 when a production request for a standard order is offered;

6 $$R_{t}^{5} = \left\{ \begin{gathered} \max \left\{ {V_{t} (T_{A} - f,T_{B} ) + \pi_{2} ,V_{t} (T_{A} ,T_{B} ) - \left. {c_{2} } \right\},T_{A} \ge f} \right. \hfill \\ \max \left\{ {V_{t} (T_{A} ,T_{B} - f) + \pi_{2} ,V_{t} (T_{A} ,T_{B} ) - \left. {c_{2} } \right\},T_{A} < f,T_{B} \ge f} \right. \hfill \\ \max \left\{ {V_{t} (T_{A} ,T_{B} ) - \left. {c_{2} } \right\},T_{A} < f,T_{B} < f} \right. \hfill \\ \end{gathered} \right.$$

and i = 6 when there is no production request.

7 $$R_{t}^{6} = V_{t} (T_{A} ,T_{B} )$$

When the production cycle ends and the production line starts on the same day, that is, T = 0, emergency orders may arrive, and the number of random emergency orders is Q. The production time of an emergency order is g time slots, and the system state is S = (T_A_,T_B_). When [T_A_/g] + [T_B_/g] > Q ([a] means the largest integer not greater than a), that is, the number of emergency orders that can be produced in the total remaining time slots is greater than the number of emergency orders actually arrived, the enterprise will incur an idle cost. Similarly, when [TA/g] + [TB/g] < Q, the enterprise will incur a corresponding rejection cost. The following expression can be obtained when TS = [TA/g] + [TB/g]:

8 $$\begin{gathered} V_{0} (T_{A} ,T_{B} ) = \pi_{3} E(T_{s} \wedge Q) - c_{4} [T_{A} + T_{B} - E(T_{s} \wedge Q) \times g] - c_{3} E(Q - T_{s} )^{ + } \hfill \\ = (\pi_{3} + c_{3} + c_{4} \times g)E(T_{s} \wedge Q) - c_{3} E(Q) - c_{4} (T_{A} + T_{B} ) \hfill \\ \end{gathered}$$

In Eq. (8), (a^b) represents min(a,b), and (a)^+^ indicates max(a,0).

### Model solution

According to the MDP model, At(T_A_,T_B_) + is the set of actions that maximize the system revenue function. According to the restriction of marginal condition Eq. (8), the existence of an optimal solution is ensured at all production times t = T, T − 1…. After reasonable parameter setting, the value iteration strategy can be employed to solve the MDP model so that the optimal decision can be made at every moment according to the number of remaining time slots and the order arrangement. This involves accepting or rejecting the production request of an order and obtaining the specific production scheduling strategy according to the set production scheduling rules.

At any moment during production, there are three conditions (a standard order, a nonstandard order, and no production request) for the order arrangements for equipment A and equipment B. Therefore, the system may exhibit six possible conditions at any moment during production (Table 2). At t = T, T − 1… when the system state is S = (T_A_,T_B_), the system compares the sizes of V_t−1_ (T_A_ + T_B_ − e) + π1 and V_t−1_ (T_A_ + T_B_) − c_1_ to determine whether the production request of a standard order should be accepted. If π_1_ + c_1_ ≥ V_t−1_ (T_A_ + T_B_) − V_t−1_ (T_A_ + T_B_ − e), the order is accepted; otherwise, it is rejected. If a nonstandard order arrives, V_t−1_ (T_A_ + T_B_ − f) + π1 is compared with V_t−1_ (T_A_ + T_B_) − c_2_, and the order will be accepted if π_2_ + c_2_ ≥ V_t−1_ (T_A_ + T_B_) − V_t−1_ (T_A_ + T_B_ − f). According to this decision rule, the system can automatically make the optimal decision when any order arrives through multiple iterations. After deciding whether to accept an order, the production scheduling strategy is generated according to the current order situation and the set production scheduling rules.

## Numerical examples

The parameters of the MDP model are set to simulate the arrangement of various orders and the optimal decision-making process of the system. The production cycle is evenly divided into 48 equal moments, namely, T = 48. At this time, the arrival time of the order and the decision moment of the system are both an any production moment t, where t = 48, 47, …, 1. The maximum numbers of available time slots for equipment A and B are both set to T = 96. The numbers of time slots for a standard order, a nonstandard order, and an emergency order are e = 4, f = 4, and g = 1, respectively. The corresponding benefits and rejection costs of the three types of orders are set to π_1_ = 200, π_2_ = 400, π_3_ = 600, c_1_ = 100, c_2_ = 300, and c_3_ = 500. In addition, the idle cost per time slot is defined as c_4_ = 100. The production request of a standard order can be given randomly at any time with a probability of λ_1_ = 0.7 and that of a nonstandard order is λ_2_ = 0.2. On the production day, the random arrival of emergency order Q follows a Poisson distribution with 20 parameters. Based on the production request arrival rate of various orders, the transition probability λ_i_ (where i = 1, 2, 3, 4, 5 and 6) can be calculated. The corresponding arrival situation and six transition probabilities are shown in Table 5.

**Table 5:** Arrival and transfer probabilities of orders.

Order arrival status	Transfer probability
Two standard order requests arrive	${\text{P}}_{1} = \lambda_{1}^{2} = 0.49$
Two nonstandard order requests arrive	${\text{P}}_{2} = \lambda_{2}^{2} = 0.04$
A standard order & a nonstandard order request arrive	P_3_ = 2λ_1_ λ_2_ = 0.28
Only a standard order request arrives	P_4_ = 2λ_1_ (1 − λ_1_ − λ_2_) = 0.14
Only a nonstandard order request arrives	P_5_ = 2λ_2_ (1 − λ_1_ − λ_2_) = 0.04
No production request arrives	P_6_ = (1 − λ_1_ − λ_2_)^2^ = 0.11

**Figure 2:**
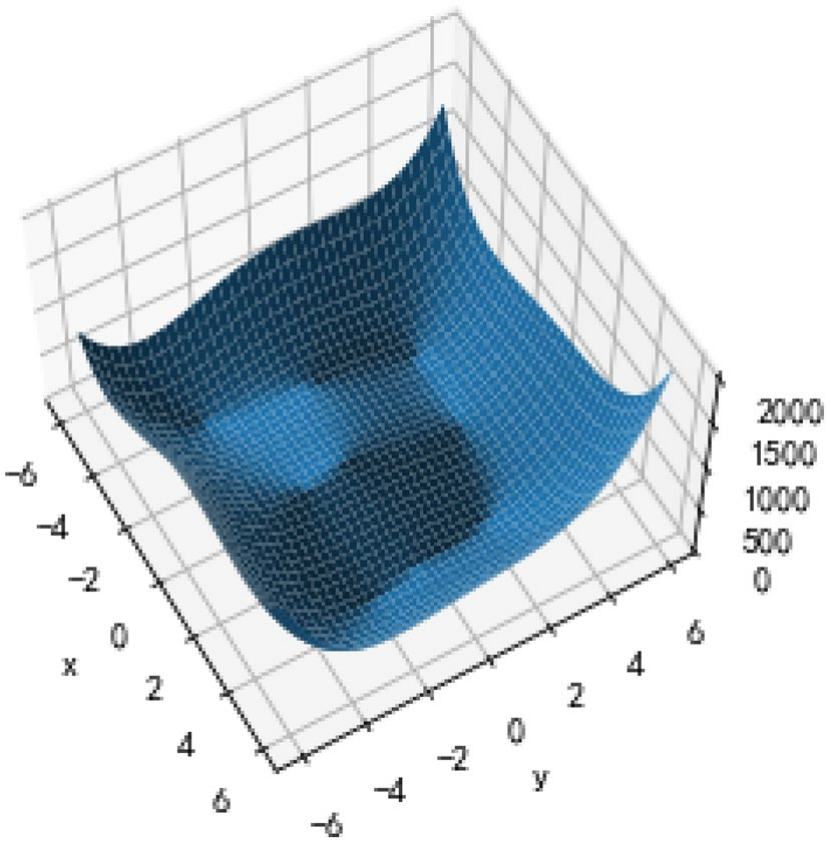
Expected revenue changes chart.

**Figure 3:**
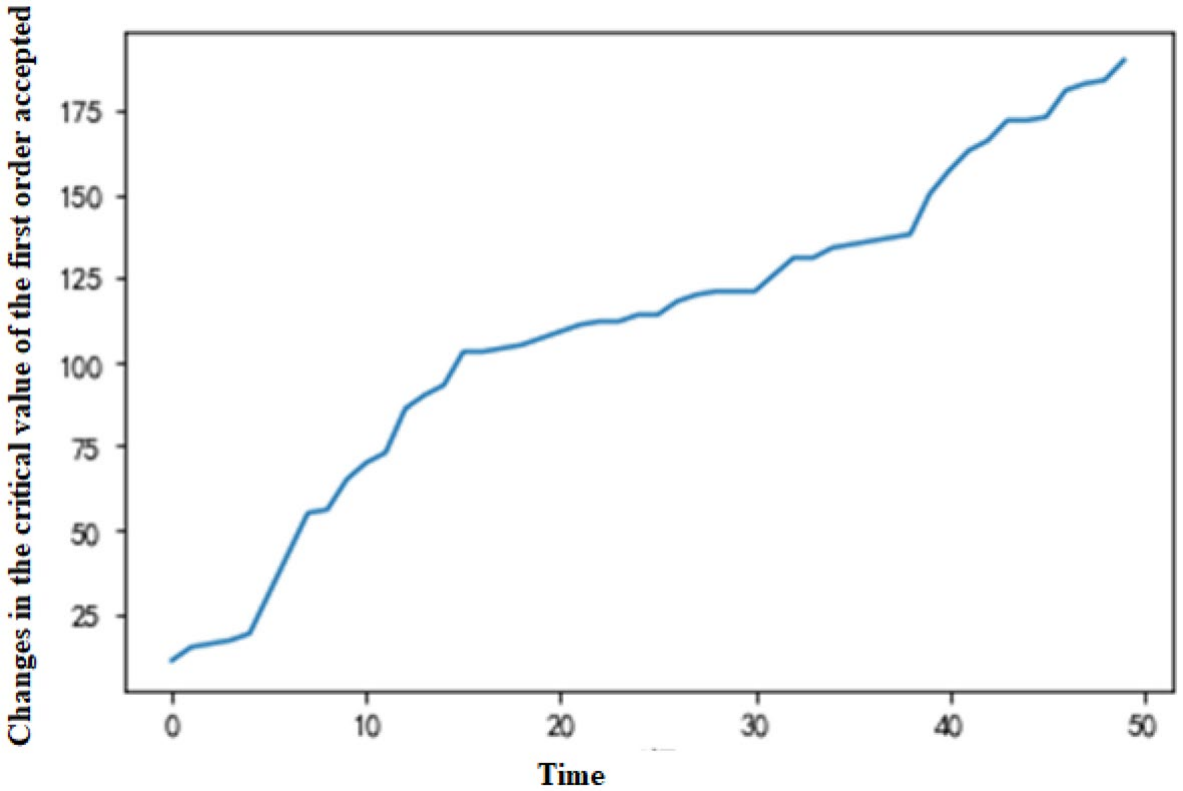
Changes in the critical value of the first order accepted.

**Figure 4:**
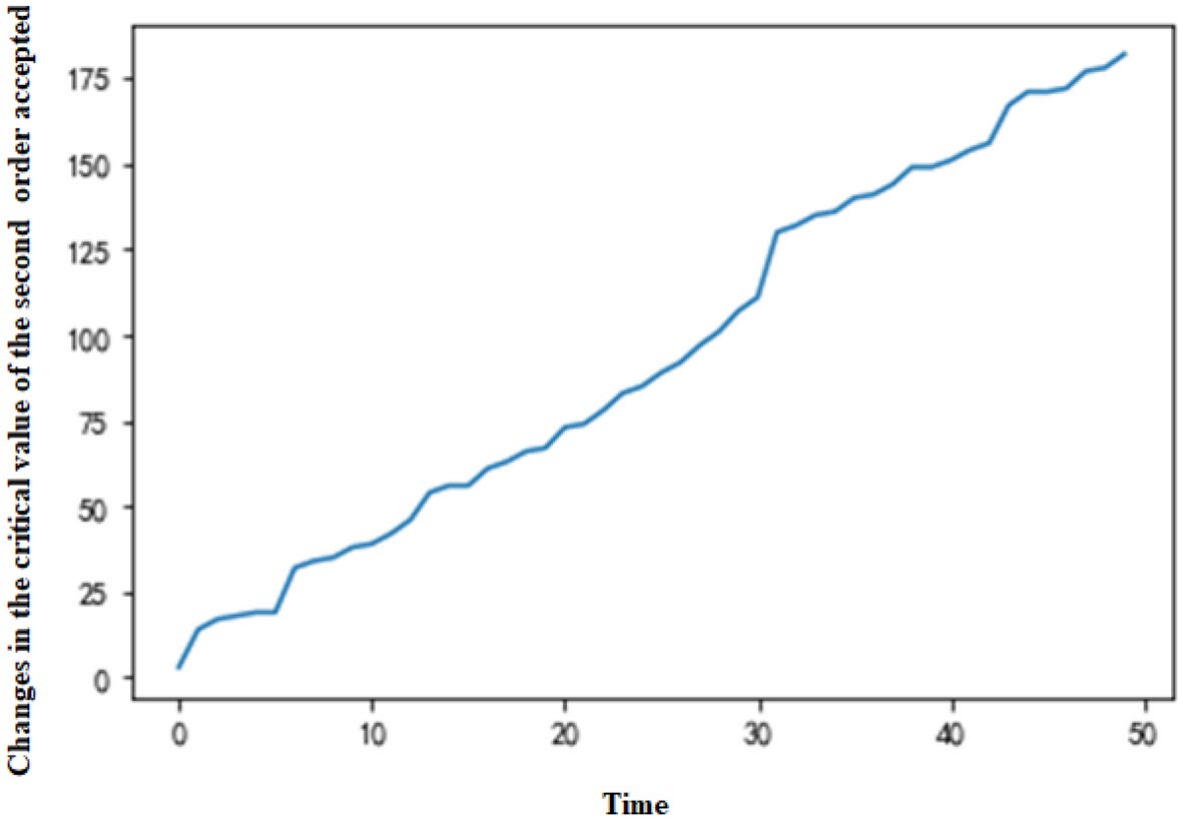
Changes in the critical value of the second order accepted.

The MDP can be simulated by Python based on the above parameter settings and dynamic programming theory. With the production request arrival rate of standard and nonstandard orders (λ_1_ and λ_2_), the arrival situation of a group in the production cycle can be simulated, and the optimal production scheduling strategy can be obtained through multilevel iteration. The example in this paper reveals that the system will generate critical values for accepting all types of orders. For a standard order, there is a critical value X at any production moment, which is the minimum value of π_1_ + c_1_ ≥ V_t−1_ (T_A_ + T_B_) − V_t−1_ (T_A_ + T_B_ − e), so the decision can be simplified as comparing T_A_ + T_B_ and X. When T_A_ + T_B_ ≥ X, π_1_ + c_1_ ≥ V_t−1_ (T_A_ + T_B_) − V_t−1_ (T_A_ + T_B_ − e), which means that the order is accepted; otherwise, it is rejected. Nonstandard orders also have a critical value. After accepting or rejecting an order, the system will arrange production based on the actual acceptance situation. If the production request of only one order is accepted, the equipment with more surplus capacity (equipment A is selected when T_A_ = T_B_) is selected for production. If the production request of a standard order or a nonstandard order is accepted, the equipment with more surplus capacity is preferentially selected for production of the nonstandard order (equipment A is selected to complete the nonstandard order if T_A_ = T_B_). If two orders of the same type are accepted, they will be assigned to equipment A and equipment B separately. The simulation results indicate that the expected revenue function Vt (T_A_ = T_B_) changes with the production time and the total number of remaining time slots, as demonstrated in Fig. 2. When production requests of two orders are entered into the system at every production moment, the changes in critical value of the system accepting the first and the second orders (a standard order or a nonstandard order) at any time are illustrated in Figs. 3 and 4, respectively.

According to the decision rules of the MDP model and the production scheduling rules, the optimal production scheduling strategy within the production cycle is shown in Table 6. In contrast, the decision-making process of the traditional production strategy is shown in Table 7. Regarding order type, 1, 2, and 3 represent a standard order production request, a nonstandard order production request, and no production request, respectively. However, in the decision-making process, 1 means the order is accepted, and 0 indicates that it is rejected. T_A_ and T_B_ represent the numbers of remaining available time slots for equipment A and equipment B at the current moment, respectively.

**Table 6:** MDP-based production scheduling strategy.

Time	Order type		Strategy		TA	TB
1	2	2	0	0	92	93
2	1	2	1	1	89	92
3	2	2	0	0	89	91
4	2	1	1	0	82	89
5	1	1	1	0	80	89
6	1	1	1	0	79	86
7	1	1	1	0	79	84
8	1	1	1	0	75	82
9	2	1	1	0	74	80
10	2	1	1	1	73	79
11	2	1	1	1	73	76
12	2	1	0	1	71	75
13	2	2	0	1	70	74
14	1	2	0	1	70	74
15	1	2	1	0	67	74
16	1	1	1	0	66	74
17	1	2	1	0	66	71
18	2	2	0	1	66	70
19	1	2	1	0	65	69
20	2	2	0	0	65	69
21	1	1	0	1	59	64
22	1	2	1	1	58	59
23	1	1	1	1	57	59
24	1	2	1	1	57	59
25	2	2	0	0	57	58
26	1	2	0	1	49	56
27	2	1	0	1	48	49
28	1	2	0	1	47	49
29	1	2	1	0	46	48
30	1	2	0	0	41	47
31	1	1	1	1	36	46
32	1	1	0	1	32	45
33	2	2	1	1	32	40
34	2	1	1	1	29	39
35	2	2	1	1	28	38
36	2	2	0	1	26	34
37	1	1	0	1	25	33
38	2	1	0	1	24	32
39	2	1	1	0	24	28
40	1	2	0	0	20	27
41	2	1	0	0	16	24
42	2	2	0	1	16	24
43	1	1	1	1	13	21
44	1	2	0	1	13	19
45	2	2	1	0	13	17
46	1	1	0	0	11	16
47	2	2	0	1	10	15
48	1	2	1	0	10	15
49	2	2	1	1	9	12
50	2	2	1	1	8	8

**Table 7:** Traditional production scheduling strategy.

Time	Order type		Strategy		TA	TB
1	1	1	1	0	98	95
2	1	1	1	0	98	95
3	1	1	1	1	86	93
4	1	2	1	1	85	91
5	2	1	0	1	82	89
6	2	2	0	1	79	88
7	1	2	1	0	79	83
8	1	1	0	1	79	82
9	2	1	1	0	79	77
10	2	2	0	0	77	75
11	2	2	0	1	74	73
12	2	2	0	0	74	65
13	1	1	1	0	72	64
14	2	2	0	1	65	64
15	2	2	1	0	62	59
16	1	2	0	0	62	56
17	1	1	0	1	59	55
18	2	1	0	1	58	55
19	2	2	0	0	57	54
20	1	2	0	0	56	53
21	1	2	1	1	54	51
22	1	2	0	1	52	49
23	1	2	0	1	51	46
24	1	2	1	0	47	41
25	2	2	1	0	45	38
26	1	1	0	1	44	38
27	1	1	0	1	44	37
28	2	2	0	0	44	36
29	1	2	0	0	42	35
30	1	1	1	1	41	33
31	1	1	1	1	41	31
32	2	2	0	0	40	29
33	2	1	0	1	39	28
34	1	2	1	0	36	28
35	2	2	1	0	35	26
36	2	2	0	0	34	25
37	1	1	1	1	34	23
38	1	2	1	1	32	22
39	1	2	0	0	31	17
40	1	1	0	1	30	17
41	2	2	0	0	25	13
42	2	2	0	0	25	12
43	2	1	1	0	22	12
44	2	1	1	0	22	12
45	2	1	0	0	18	11
46	1	2	0	1	11	10
47	1	2	0	1	10	10
48	2	2	0	0	9	9
49	2	2	0	0	8	8
50	2	2	1	0	7	7

**Figure 5:**
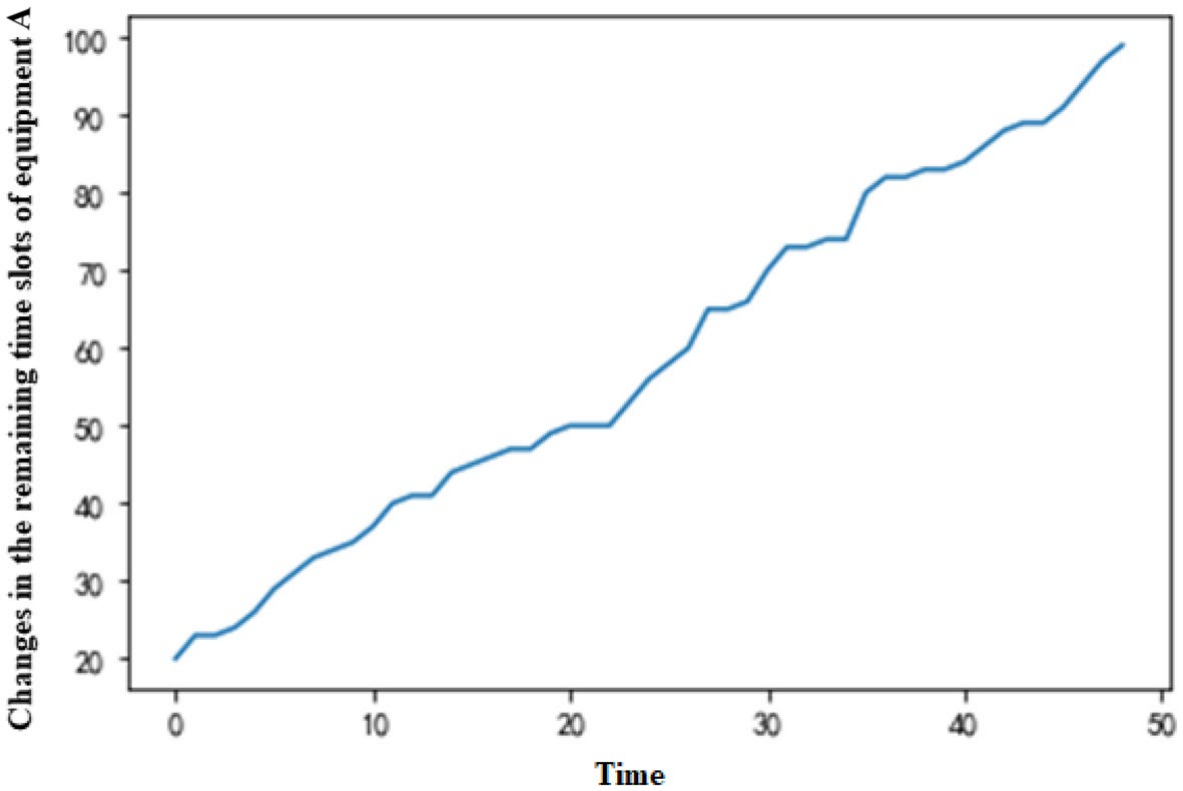
Changes in the remaining time slots of equipment A.

**Figure 6:**
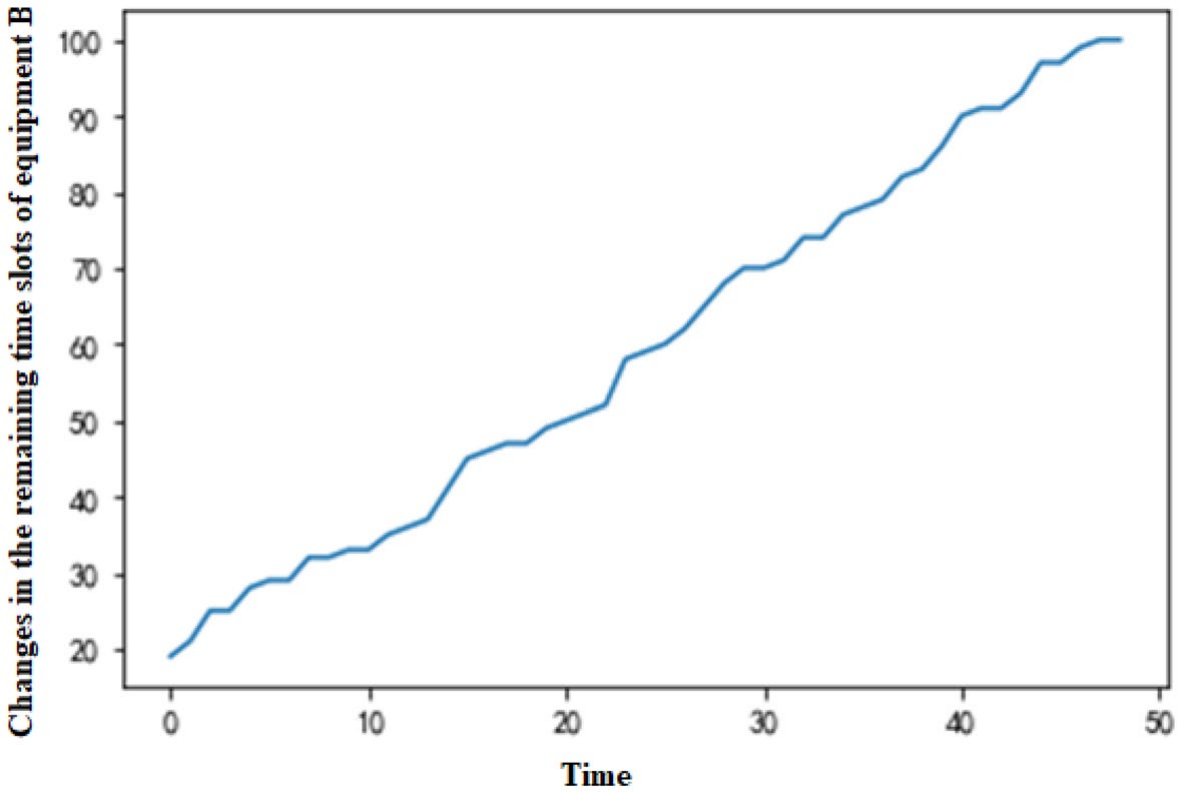
Changes in the remaining time slots of equipment B.

In the decision-making process of the MDP, the numbers of remaining available time slots of the two equipment types change with the production time (Figs. 5 and 6), which corresponds to the production scheduling strategy on the production day. Since the equipment with more surplus capacity is preferentially selected for order production at any production moment, the change in the number of remaining time slots is similar for equipment A and B. In this way, this approach balances the utilization of the production capacity and conforms to the actual equipment situation in simultaneous production on the day of production.

Based on the production strategy in this paper, the maximum total revenue of the system in the production cycle is 19,600, while that of the traditional FCFS is 17,000. Therefore, the scheduling strategy adopted in this paper exhibits better scheduling ability.

## Sensitivity analysis

### Change in the maximum number of available time slots T for the equipment

**Figure 7:**
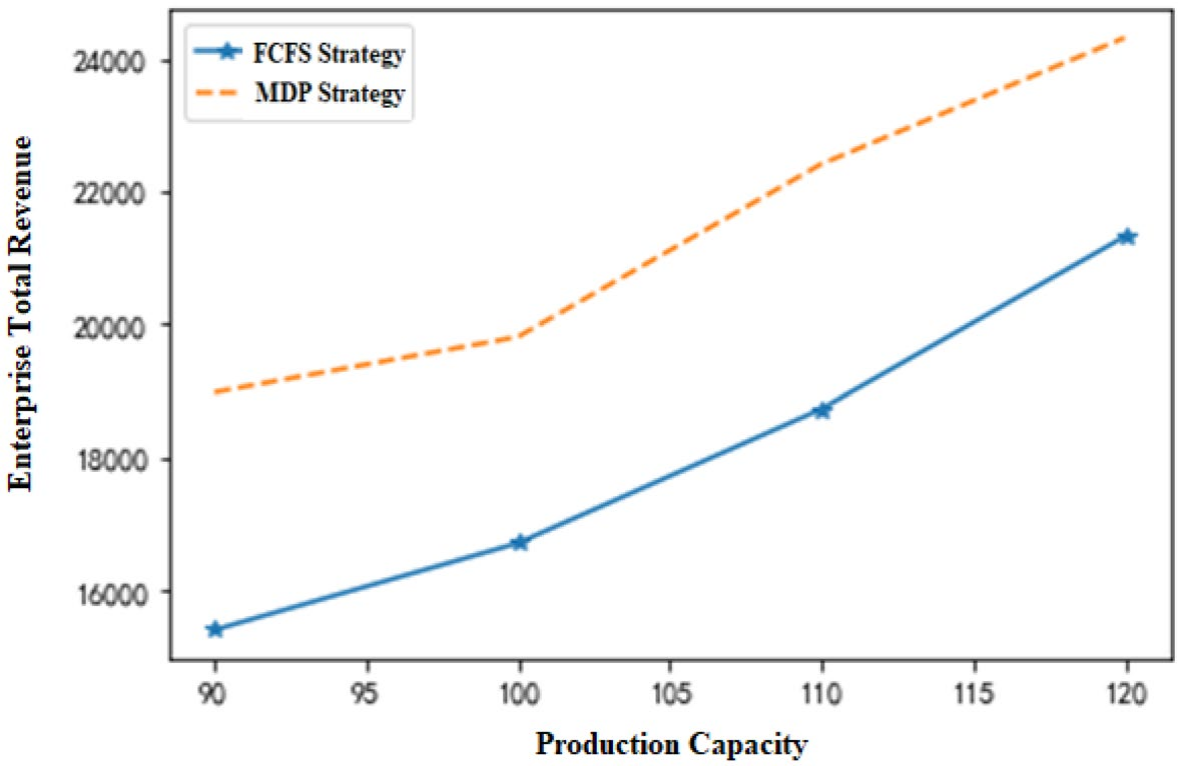
Changes in enterprise revenue with production capacity.

**Figure 8:**
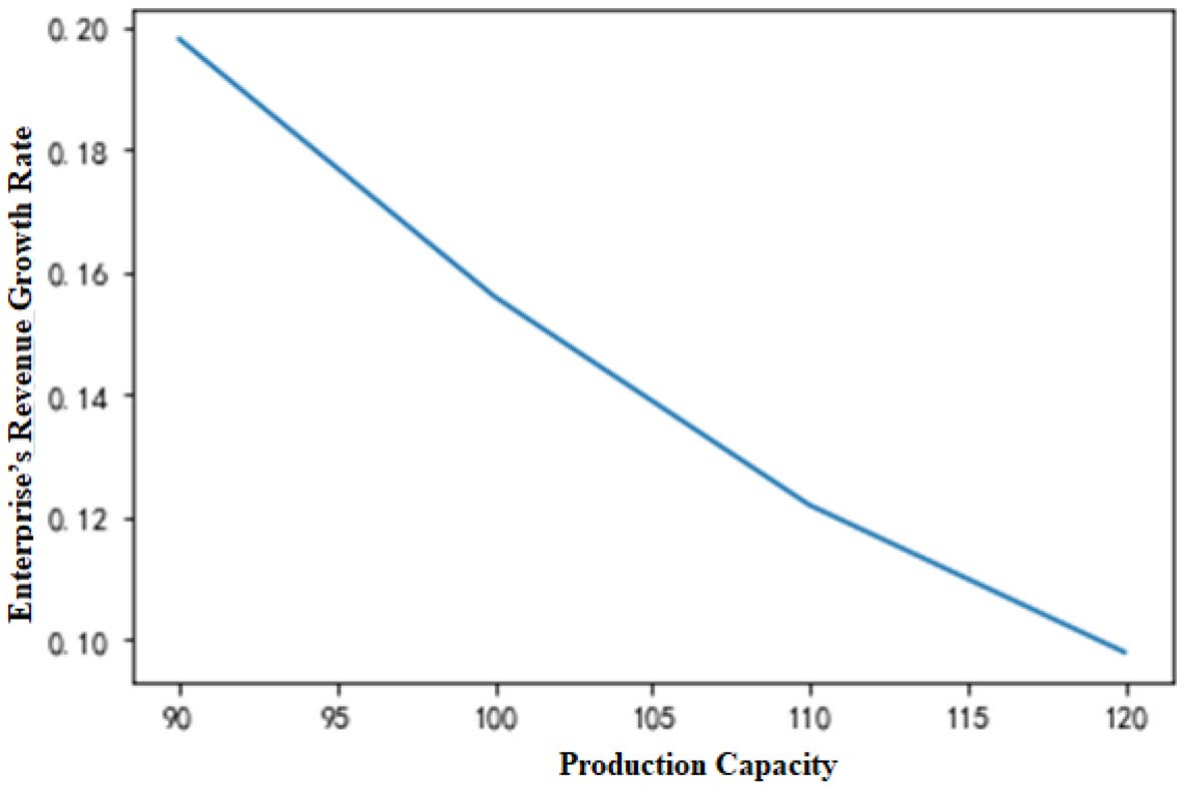
Changes in the enterprise’s revenue growth rate with production capacity.

While all other parameters remain unchanged, the maximum number of available time slots T per day for the two equipment types is changed, and the total revenue of the enterprise changes with the total capacity of the system (Fig. 7). Therefore, the following conclusions can be drawn. (1) When the total production capacities of the two pieces of equipment fail to satisfy the production requests of all orders, the total revenue increases with the number of available time slots. (2) The smaller the number of available time slots is, the lower the capacity of the production system, and the more obvious the advantage of the MDP production scheduling strategy over the traditional production scheduling strategy. This result is better illustrated by the changes in the total revenue growth rate of the enterprise with capacity (Fig. 8). It is assumed in the figure that the MDP production strategy yields total income Y_1_, while the traditional production strategy yields total income Y_2_. Meanwhile, β indicates the total income growth rate of the enterprise.

9 $$\beta = \frac{{Y_{1} - Y_{2} }}{{Y_{2} }}$$

### Change in the production request arrival rate of nonstandard orders λ_2_

**Figure 9:**
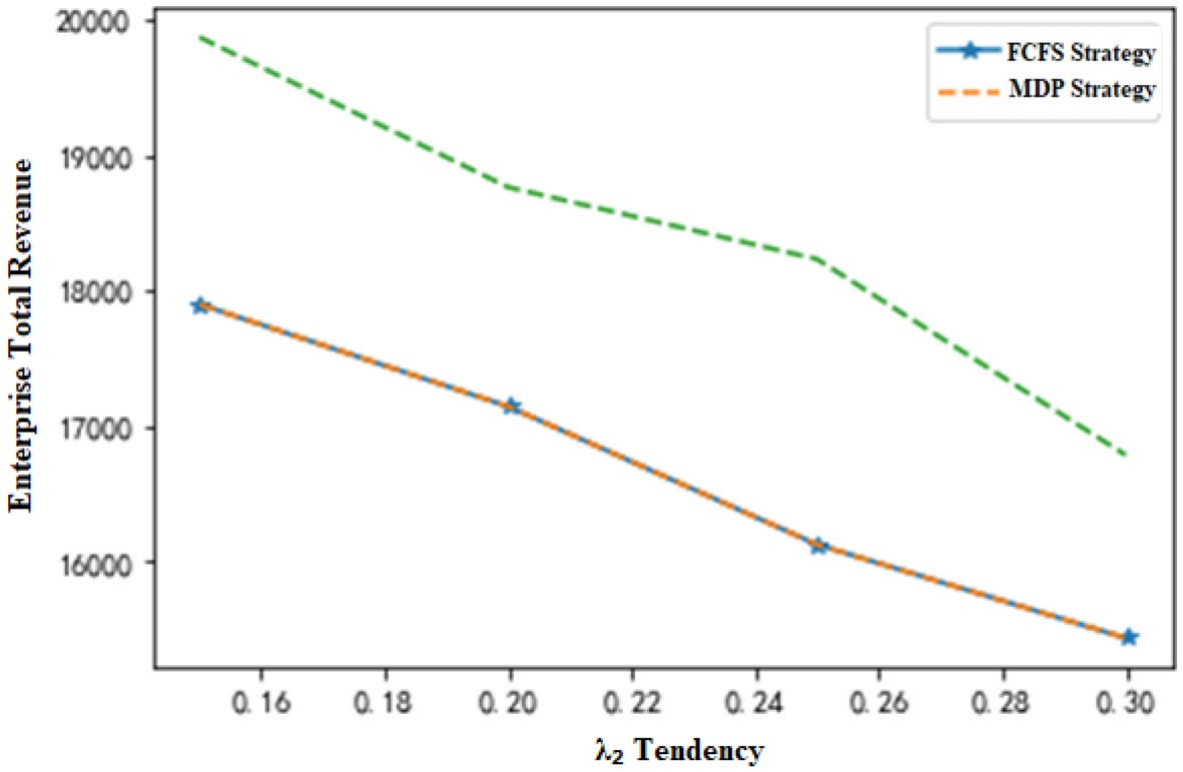
Changes in total enterprise revenue with λ_2_.

**Figure 10:**
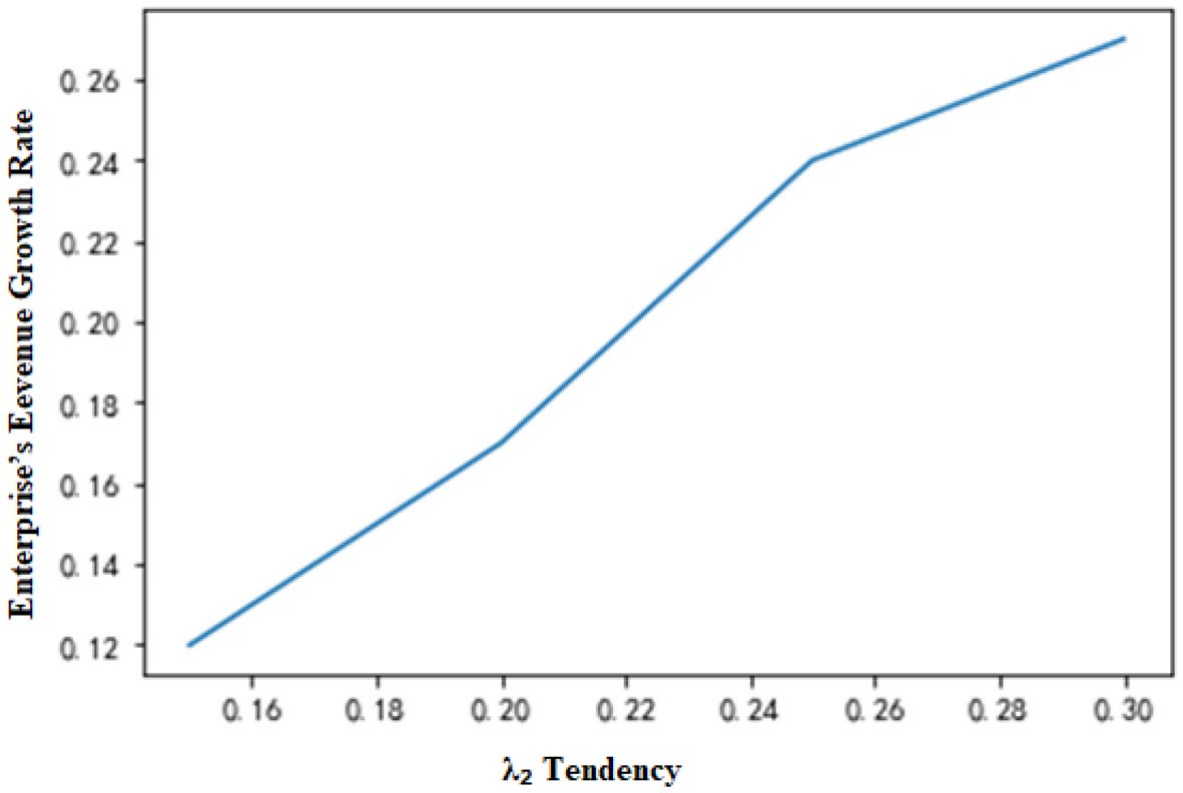
Change chart of enterprise’s revenue growth rate with λ_2_.

When the other conditions remain unchanged and the production request arrival rate of nonstandard orders is λ_2_, the changes in total revenue of the enterprise with λ_2_ can be demonstrated, as shown in Fig. 9. If the total production capacity of the system is insufficient for all the required production requests of orders, the following conclusions can be drawn. (1) As the production request arrival rate of nonstandard orders increases, the total revenue of the enterprise changes little under the MDP-based strategy but presents a decreasing trend under the traditional production scheduling strategy. This is because in the case of a shortage of production capacity, enterprises will reject more orders with an increase in the production request arrival rate of nonstandard orders, increasing the rejection cost. Under the MDP-based production scheduling strategy, enterprises can complete more nonstandard orders with higher comprehensive income, achieving a balance of total income. (2) As the production request arrival rate of nonstandard orders increases, the revenue growth rate of the MDP-based strategy shows an upward trend when compared with that of the traditional production scheduling strategy (Fig. 10). Since the comprehensive income of nonstandard orders is higher than that of standard orders (i.e., π_2_ + C_2_ ≥ π_1_ + C_1_), the MDP-based production scheduling strategy adjusts the critical value of order acceptance according to the change in the production request arrival rate of nonstandard orders to retain the capacity for later arriving nonstandard orders. On the other hand, more production requests for nonstandard orders will result in the rejection of more later arriving nonstandard orders under the FCFS, increasing the rejection cost.

In general, if the other variables remain unchanged, when the maximum number of available time slots (T) for each piece of equipment changes, the indicators under the two production scheduling strategies change with the total production capacity, as shown in Table 8. In the table, the value at T = 80 is used as the initial benchmark, and the change value of each indicator reflects the change in the next state based on the previous state. That is, T = 90 corresponds to the changes at T = 80, and T = 100 corresponds to the changes at T = 90 until T = 130. Under the condition of other variables remaining unchanged, the production arrival rate λ_2_ of nonstandard orders changes when the index changes, as displayed in Fig. 7. λ_2_ = 0.1 is defined as the initial base, and the analogy is the same as that given in Table 8, A change in the next state based on the previous state is also observed. Based on Tables 8 and 9, it can be concluded that the lower the system capacity and the higher the arrival rate of nonstandard orders are, the more prominent the superiority of the MDP-based production scheduling strategy over the traditional production scheduling strategy.

**Table 8:** Changes in various indicators with production capacity.

A equipment time slot	FCFS income change	MDP income change	Change of income growth rate
80	12122	15989	0.233
90	15412	18982	0.198
100	16716	19812	0.156
110	18732	22412	0.122
120	21321	24312	0.098
130	24565	25187	0.055

The calculation was rounded up.

**Table 9:** Changes in various indicators with λ_2_.

λ_2_	FCFS income change	MDP income change	Change of income growth rate
0.1	19123	22312	0.08
0.15	17898	19872	0.12
0.2	17143	18763	0.17
0.25	16123	18232	0.24
0.3	15432	16784	0.27
0.35	15112	16332	0.32

The calculation was rounded up.

## Conclusions

This paper proposes an MDP-based strategy to dynamically process the production scheduling of orders in manufacturing enterprises and to optimize the objectives to maximize the benefits of production equipment. With two types of production equipment and three types of orders with different production times, the optimal production scheduling strategy of the system is analyzed via dynamic programming theory. After reasonable parameter setting, multistage iteration is performed with Python. The simulation results prove that compared with the traditional FCFS, the MDP-based production scheduling strategy adopted in this paper exhibits several advantages, which can maximize the benefits in terms of the production system of manufacturing enterprises. Then, based on the results of the sensitivity analysis, the maximum numbers of available time slots and the arrival rate λ_2_ of nonstandard orders of the two pieces of equipment are changed for comparative analysis. The results indicate that the MDP-based strategy is suitable for the production scheduling of manufacturing enterprises. The MDP model is superior to the traditional production decision-making model when the system capacity is insufficient and the arrival rate of nonstandard orders is high. Follow-up research can be conducted based on consideration of the difference between the service capacity and efficiency of different equipment and analysis of other parameters that influence the enterprise revenue rate. In addition, factors such as inventory thresholds that affect the on-time delivery rate of orders can be considered in the model.

## Data Availability

The datasets generated during the current study are available from the corresponding author on reasonable request.

## References

[1] Zhong RY, Xu X, Klotz E, Newman ST (2017). Intelligent manufacturing in the context of industry 4.0: A review. Engineering.

[2] Zhou J, Li P, Zhou Y, Wang B, Zang J, Meng L (2018). Toward new-generation intelligent manufacturing. Engineering.

[3] Young, L.C. *The Application of Orthogonal Collocation to Laminar Flow Heat and Mass Transfer in Monolith Converters*, MSD Thesis (University of Washington, 1974).

[4] Han G, Gao T, Wang L (2011). Research on measurement, volatility and causes of excess production capacity of Chinese manufacturing industries. Econ. Res. J..

[5] Manavizadeh N, Tavakoli L, Rabbani M (2013). A multi-objective mixed-model assembly line sequencing problem in order to minimize total costs in a Make-To-Order environment, considering order priority. J. Manuf. Syst..

[6] Liu WH, Xu XC, Ren ZX (2011). An emergency order allocation model based on multi-provider in two-echelon logistics service supply chain. Supply Chain Manag. Int. J..

[7] Trzyna D, Kuyumcu A, Lödding H (2012). Throughput time characteristics of rush orders and their impact on standard orders. Procedia CIRP.

[8] He XM, Dong SH, Zhao N (2020). Research on rush order insertion rescheduling problem under hybrid flow shop based on NSGA-III. Int. J. Prod. Res..

[9] Gupta YP (1990). A feasibility study of JIT purchasing implementation in a manufacturing facility. Int. J. Oper. Prod..

[10] Golhar DY, Sarker BR (1992). Economic manufacturing quantity in a just-in-time delivery system. Int. J. Prod. Res..

[11] Caramanis C, Anli OM (1999). Dynamic lead time modeling for JIT production planning. Pro. IEEE Int. Conf. Robot. Autom..

[12] Ching, W. K., & Ng, M. K. *Markov Chains: Models, Algorithms and Applications* (Springer, 2020).

[13] Nie L, Shao X, Gao L, Li W (2010). Evolving scheduling rules with gene expression programming for dynamic single-machine scheduling problems. Int. J. Adv. Manuf. Technol..

[14] Fattahi P, Fallahi A (2010). Dynamic scheduling in flexible job shop systems by considering simultaneously efficiency and stability. CIRP J. Manuf. Sci. Technol..

[15] Vinod V, Sridharan R (2011). Simulation modeling and analysis of due-date assignment methods and scheduling decision rules in a dynamic job shop production system. Int. J. Prod. Econ..

[16] Pickardt CW, Hildebrandt T, Branke J, Heger J, Scholz-Reiter B (2013). Evolutionary generation of dispatching rule sets for complex dynamic scheduling problems. Int. J. Prod. Econ..

[17] Hamzadayi A, Yildiz G (2016). Event driven strategy based complete rescheduling approaches for dynamic identical parallel machines scheduling problem with a common server. Comput. Ind. Eng..

[18] Rajabinasab A, Mansour S (2011). Dynamic flexible job shop scheduling with alternative process plans: an anent-based approach. Int. J. Adv. Manuf. Technol..

[19] Zhou L, Zhang L, Sarker BR (2018). An event-triggered dynamic scheduling method for randomly arriving tasks in cloud manufacturing. Int. J. Comput. Integr. Manuf..

[20] Wei W, Mao L, Li W (2010). The dynamic optimal method of emergency resources development planning based on markov decision processes. Acta Scientiarum Naturalium Universitatis.

[21] Qian J, Wu K (2022). Optimal order acceptance decision based on after-state reinforcement learning. Comput. Sci..

